# Digital Humans for Depression Assessment and Intervention Support: Scoping Review

**DOI:** 10.2196/79954

**Published:** 2026-01-05

**Authors:** Jiashuo Cao, Wujie Gao, Ruoyu Wen, Chen Li, Simon Hoermann, Nilufar Baghaei, Mark Billinghurst

**Affiliations:** 1 Auckland Bioengineering Institute, University of Auckland, Auckland, New Zealand; 2 Department of Applied Social Sciences, The Hong Kong Polytechnic University, Hong Kong, China (Hong Kong); 3 School of Product Design, University of Canterbury, Christchurch, New Zealand; 4 Department of Computing, The Hong Kong Polytechnic University, Hong Kong, China (Hong Kong); 5 School of Electrical Engineering and Computer Science, The University of Queensland, Brisbane, Australia

**Keywords:** digital human, virtual agent, mental health, depression, embodied conversational agent

## Abstract

**Background:**

The growing global burden of mental health disorders has intensified the search for scalable, accessible, and cost-effective interventions. Conversational agents in the form of digital humans have emerged as promising tools to deliver mental health support across diverse populations and settings.

**Objective:**

This scoping review aimed to analyze the role of digital humans in depression management, identifying their specific applications in both diagnostic processes and therapeutic interventions. Additionally, it aimed to evaluate the design choices implemented in digital human systems, including their appearance, interaction modalities, back-end intelligence systems, and the various roles they assume.

**Methods:**

Following the PRISMA-ScR (Preferred Reporting Items for Systematic Reviews and Meta-Analyses extension for Scoping Reviews) guidelines, we systematically searched peer-reviewed literature across major databases, including ACM Digital Library, IEEE Xplore, Web of Science, and PubMed, to capture both psychological and technological perspectives. The search query included a wide variety of synonyms for digital humans and depression: (“avatar” OR “virtual agent” OR “embodied conversational agent” OR “relational agent” OR “digital human” OR “virtual human” OR “virtual character”) AND (“Major Depressive Disorder” OR “Depression”). Studies were included if they described the development, implementation, or evaluation of digital humans designed to support mental health outcomes. Data were charted on agent design, therapeutic approach, target population, delivery context, and reported effectiveness.

**Results:**

In total, 20 studies (2010‐2024) were included. Depression assessment studies comprised 35% (n=7), interventions 55% (n=11), and combined approaches 10% (n=2). Assessment protocols included the questionnaires Patient Health Questionnaire-9 and Very Short Visual Analog Scale of the Center for Epidemiologic Studies Depression Scale - Visual Analog Scale - Very Short version, semistructured interviews based on *Diagnostic and Statistical Manual of Mental Disorders, Fifth Edition* criteria, and interactive tasks designed to elicit emotional responses. Intervention approaches used cognitive behavioral therapy, psychoeducation, compassion-focused therapy, and avatar therapy. Digital humans assumed 5 distinct roles: interviewer (n=6), facilitator (n=3), counselor (n=3), educator (n=3), and actor (n=5). Interviewers primarily appeared in assessment studies, presenting structured questions. Counselors engaged in therapeutic dialogues, while educators delivered psychoeducational content. Facilitators assisted participants in achieving system goals. Actors portrayed specific emotions or dysfunctional beliefs to facilitate therapeutic processes. Studies highlighted digital humans’ utility in enhancing diagnostic processes and therapeutic interventions, noting the potential for transformation through physiological data integration.

**Conclusions:**

This study demonstrates that digital humans represent a transformative advancement in depression management, offering innovative applications across both assessment and intervention phases. The evidence reveals digital humans’ effectiveness in replicating traditional therapeutic roles while providing unique advantages, including 24/7 accessibility, reduced stigma, consistent care delivery, and personalized support. Digital humans can successfully function to establish therapeutic alliances and elicit meaningful engagement comparable with human providers. Findings underscore the need for continued research to fully realize digital humans’ potential in addressing depression-specific needs, advocating for expansion into diverse therapeutic scenarios, and exploration of unexplored digital human applications.

## Introduction

Depression is a prevalent mental health disorder affecting millions of individuals worldwide, with the World Health Organization identifying it as a leading cause of disability globally [1]. Characterized by persistent sadness, lack of interest in daily activities, and a multitude of physical and psychological symptoms, depression can severely impact an individual’s quality of life [2]. Psychotherapy’s effectiveness in treating depression across diverse populations and settings is widely recognized [3,4]. It has been demonstrated to be as effective as antidepressant medication for individuals with mild to moderate depression and is often the preferred initial treatment option for patients [5,6]. Furthermore, psychotherapy may surpass pharmacological treatment of depression in terms of long-term effectiveness [7].

In recent years, technology has been integrated into mental health therapy, allowing for novel ways of therapy as well as reaching people who otherwise would not have access to therapy [8]. One of those recent technology advancements is embodied conversational agents, often in the form of virtual human characters—in this paper referred to as digital humans [9]—which have shown potential for health assessments and interventions [10].

Hence, the emergence of virtual characters as a supportive tool for depression highlights a significant trend, which is propelled by advancements in artificial intelligence and computer graphics [11]. These digital characters provide increasingly lifelike, responsive, and immersive interactions, capable of perceiving and reacting to the emotional states of users [9]. They offer customized support and interventions, paralleling the capabilities of human therapists to a notable extent [12]. This technological progression enables the delivery of online therapy exercises, mindfulness techniques, and emotional support accessible for a wide range of people. Individuals can engage with these therapeutic resources from their own homes, effectively overcoming obstacles such as societal stigma, geographic barriers, and the prohibitive costs often associated with traditional therapeutic services [13,14]. Moreover, the incorporation of machine learning algorithms allows these virtual agents to evolve through user interaction, enhancing their supportive capabilities over time and furnishing a personalized therapeutic experience [15]. As these technological innovations advance, virtual characters are poised to become a fundamental component of mental health care. Based on current literature, while there have been reviews and meta-analyses examining the use of digital humans and digital interventions in health contexts, a critical gap exists in understanding how digital humans have been specifically used to support depression and what detailed design choices have been made to adapt them for this purpose. For instance, Ma et al [11] conducted a meta-analysis of virtual human interventions across various health conditions, providing valuable insights into intervention outcomes, but did not focus specifically on digital humans in depression contexts nor examine the design characteristics that enable therapeutic interactions. Chattopadhyay et al [12] explored the application of virtual humans in health care systems broadly, emphasizing implementation contexts and user perceptions, but did not analyze the technical and aesthetic design decisions—such as appearance choices, interaction modalities, or behavioral capabilities—that shape these systems. Moshe et al [14] discussed the effectiveness of digital interventions for depression but focused primarily on app-based and online platforms, lacking in-depth exploration of embodied conversational agents and their unique design considerations. Thus, a comprehensive analysis of digital humans’ roles in depression management, revealing their exact value, is needed. To fulfill the goal of unpacking the benefits of using digital humans to support depression, we investigate two research questions (RQs):

- First, how are digital humans used in the assessment and intervention of depression?
- Second, what design considerations were made to adapt digital humans for depression assessment and intervention?

This study presents 2 primary contributions. First, it provides a detailed overview of the current state of research regarding the use of digital humans in supporting individuals with depression. This encompasses a thorough analysis of the various types of support services available and the specific design choices regarding the implementation of digital humans. Second, it identifies and proposes several areas for future research within this domain that merit further investigation.

## Methods

### Overview

We conducted the scoping review in February 2025. Scoping reviews aim to facilitate the formulation of pertinent RQs by rapidly identifying and categorizing existing evidence within a given field [16]. Our methodology was anchored in the guidelines set forth by Munn et al [17], complemented by the PRISMA (Preferred Reporting Items for Systematic Reviews and Meta-Analyses) checklist’s extension, specifically the PRISMA-ScR (Preferred Reporting Items for Systematic Reviews and Meta-Analyses extension for Scoping Reviews) framework [18], which is designed for scoping reviews.

### Definitions

#### Digital Human

In this review, the definition of digital human is equivalent to the definition of virtual human. As described by Traum [19], a virtual human is an “artificial agent that includes both a visual body with a humanlike appearance and range of observable behaviors, and a cognitive component that can make decisions and control the behaviors to engage in human-like activities.” Although this definition is comprehensive, there are still some ambiguities, such as the judgment of humanlike appearance and humanlike activities. To provide a clearer set of criteria to assist us in filtering articles, we have defined a digital human as an agent with 3 criteria inspired by the 10 traits suggested by Burden and Savin-Baden [9]. A digital human (1) visually possesses realistic appearance characteristics of a human, including facial features and body proportions; (2) is capable of performing nonverbal behaviors, including body movements and facial expressions; and (3) must engage in bidirectional communication, understanding, and responding to verbal and nonverbal cues from users.

If the virtual collocutor described in the reviewed article meets all of the criteria, then we consider it to be a digital human.

#### Support for Depression

Support for depression encompasses a holistic approach that integrates both direct and indirect methods to aid individuals in managing and overcoming the condition. This comprehensive support system is essential for addressing the multifaceted nature of depression, which affects individuals emotionally, physically, and socially [3]. Following the framework established by Cuijpers [20], we distinguish between direct and indirect support based on their primary target. Indirect support focuses on problems related to depression—such as social isolation, lifestyle factors, or caregiver burden—where interventions address associated factors rather than depression as the primary clinical target. In contrast, direct support explicitly targets depression assessment (eg, administering Patient Health Questionnaire-9 [PHQ-9], conducting diagnostic interviews based on *Diagnostic and Statistical Manual of Mental Disorders, Fifth Edition* [*DSM-5*] criteria) or depression-specific therapeutic interventions (eg, cognitive behavioral therapy [CBT] for negative thought patterns and compassion-focused therapy [CFT] for self-criticism). In this paper, we narrow the scope to direct support provided to individuals with depression, as this aligns with our RQs that specifically examine how digital humans function within depression assessment and intervention protocols. This means that the primary contribution of selected papers is to provide assistance to individuals undergoing assessment for depression and receiving treatment for it.

### Information Sources and Search Strategy

Given the interdisciplinary nature of the topic, the search was carried out across four distinct digital libraries, spanning both psychological and technological fields: ACM Digital Library, IEEE Xplore, Web of Science, and PubMed. Based on the RQs and definition of digital human, we constructed the search query “(“avatar” OR “virtual agent” OR “embodied conversational agent” OR “relational agent” OR “digital human” OR “virtual human” OR “virtual character”) AND (“Major Depressive Disorder” OR “Depression”).” We did not include context-related terms in the search query (such as assessment, screening, and intervention) because we aim to find as many records as possible of digital humans supporting depression. Later, we will filter out the articles that meet the requirements of this paper using exclusion criteria.

### Eligibility Criteria

Based on our definition of digital human and RQs, we created some inclusion and exclusion criteria (Textbox 1). The exclusion criteria were used to remove papers from consideration.

Textbox 1. **Inclusion and exclusion criteria.**

**Inclusion criteria**

- Full text available.
- Paper must be published in English.
- Paper provides details of digital human.
- At least one empirical study has been conducted on depression.
- The purpose of the study is to support depression assessment or intervention.
- The study included interaction between participants and digital humans.

**Exclusion criteria**

The measured outcome is not related to depression or major depressive disorder.

- The contribution of work does not support depression assessment or intervention.
- No digital human described in the paper.
- No interaction between participant and digital human.

### Search Result and Study Selection

The search led to the identification of 1031 publications. After excluding duplicate records, we amassed a total of 909 papers. The assessment process for these papers was conducted in 2 distinct phases. Initially, a preliminary assessment based on titles and abstracts was carried out, with the first 50 papers being collaboratively reviewed by the first, second, and third authors (JC, WG, and RW). Following a consensus on the inclusion and exclusion decisions regarding these 50 papers, the remaining papers were evenly distributed among the same 3 authors for review. This first phase resulted in the identification of 52 pertinent papers. Subsequently, 3 additional publications were manually sourced, culminating in a total of 55 new references. These references were then apportioned among 4 authors (JC, WG, RW, and CL) for an in-depth assessment of the full texts, guided by the predefined inclusion and exclusion criteria. This process led to the selection of 20 papers [21-40] for subsequent data extraction.

### Data Charting Process and Data Items

Data extraction was carried out by the first and second authors (JC and WG), ensuring a thorough and collaborative approach to gathering information. This process involved detailing the characteristics of each study. To facilitate coordination and accuracy, the extracted data were compiled into a shared Google Sheet. To further enhance the reliability of the data collection process, the first author (JC) conducted a comprehensive review of all data extractions, ensuring consistency and accuracy across the dataset. Specifically, the following data items were extracted from selected papers:

- General characteristics: title, authors, year of publication, journal or proceedings, and study aims (Table 1).
- Study design and findings: setting, sample size, protocol, role of digital human, and findings (Table 2).
- Digital human design: appearance, display device, system type, and input and output modality (Table 2).

**Table 1.** Summary of selected studies.

Study	Journal or proceedings	Experiment settings	Participants	Service	Aim of study
Jaiswal et al [21]	19th ACM^a^ International Conference on Intelligent Virtual Agents	Laboratory	n=55	Assessment	Investigated digital human administered questionnaires for depression and anxiety
Egede et al [22]	21st ACM International Conference on Intelligent Virtual Agents	Laboratory	n=56	Assessment	Evaluated the effectiveness of digital human-mediated tasks in depression assessment
Wolters et al [23]	HCI^b^ KOREA 2015	Field	n=4	Assessment and intervention	Explored the use of personal monitoring system with digital human integrated
Baghaei et al [24]	2021 ACM SIGCHI^c^ Symposium on Engineering Interactive Computing Systems	Laboratory	n=23	Intervention	Investigated the feasibility of CFT^d^ with digital human in VR^e^
Luerssen and Hawke [25]	18th International Conference on Intelligent Virtual Agents	Field	n=9	Intervention	Evaluated the effectiveness of LiCBT^f^ conducted by virtual coach
Takemoto et al [26]	Journal of Eye Movement Research	Laboratory	n=27	Assessment	Explored the possibility of using digital human communication and eye tracking to detect depression
Bresó et al [27]	Expert Systems	Laboratory	n=60	Intervention	Evaluated the usability and acceptability of a digital human that could identify and provide an early intervention for depression
Takemoto et al [28]	Frontiers in Digital Health	Laboratory	n=27	Assessment	Explored the possibility of using digital human communication and facial expression to detect depression
DeVault et al [29]	14th annual SIGdial^g^ Meeting on Discourse and Dialog	Laboratory	n=43	Assessment	Explored the presence of indicators of psychological distress in semi-structured digital human interview
Ashrafi et al [30]	International Conference on Human-Computer Interaction 2024	Laboratory	n=22	Intervention	Explored the effect of visual similarity of digital human in psychotherapy
Wu et al [31]	IEEE^h^ Transactions on Affective Computing	Laboratory	n=168	Assessment	Evaluated the accuracy of using digital human for automatic depression-level stratification on mobile devices
Kocur et al [32]	Frontiers in Psychiatry	Clinic	n=54	Intervention	Evaluated the effectiveness of a computer-assisted, avatar-based therapy in reducing dysfunctional beliefs in depressive inpatients
Burton et al [33]	Telemedicine and Telecare	Field	n=28	Assessment and intervention	Explored the use of personal monitoring system with digital human integrated
Shamekhi et al [34]	16th International Conference on Intelligent Virtual Agents	Field	n=20	Intervention	Investigated the use of digital human to review material after medical visit
Halim et al [35]	JMIR^i^ Mental Health	Laboratory	n=36	Intervention	Evaluated the usability and acceptability of CFT with digital human in VR
Bickmore et al [36]	Interacting with Computers	Clinic	n=131	Intervention	Explored the use of digital human explain self-care regimen to patients with depressive symptoms
Ring et al [37]	2016 ACM CHI^j^ workshop on Computing and Mental Health	Laboratory	n=10	Intervention	Investigated the efficacy of virtual therapist for depression counseling
Shamekhi et al [38]	2017 International Conference on Persuasive Technology	Field	n=154	Intervention	Evaluated the effectiveness of using digital humans for stress management
Philip et al [39]	Scientific Reports	Clinic	n=179	Assessment	Evaluated the performance and acceptability of using digital human as a diagnostic tool for depression through interview
Hidding et al [40]	Behaviour Research and Therapy	Laboratory	n=68	Intervention	Investigated the effects of a VR intervention on self-criticism and self-compassion, including the use of a digital human

a ACM: Association for Computing Machinery.

b HCI: Human-Computer Interaction.

c SIGCHI: Special Interest Group on Computer-Human Interaction.

d CFT: compassion-focused therapy.

e VR: virtual reality.

f LiCBT: low-intensity cognitive behavioral therapy.

g SIGdial: Special Interest Group on Discourse and Dialogue.

h IEEE: Institute of Electrical and Electronics Engineers.

i JMIR: Journal of Medical Internet Research.

j CHI: Conference on Human Factors in Computing Systems.

**Table 2.** Details of digital human in selected studies.

Study	Protocol	Role of digital human	Appearance	Input modality	Output modality	Back-end intelligence
Jaiswal et al [21]	Interview	Interviewer	Full body	Speech	Text, speech, behavior	Rule-based
Egede et al [22]	Computer-based interactive task	Facilitator	Full body	Speech	Text, speech, behavior	Scripted
Wolters et al [23]	PHQ-9^a^, CBT^b^	Facilitator	Upper body	Text and speech	Speech, behavior	Scripted
Baghaei et al [24]	CFT^c^	Actor	Full body	Speech	Speech, behavior	Scripted
Luerssen and Hawke [25]	LiCBT^d^	Counselor	Upper body	Text and speech	Text, speech, behavior	Rule-based
Takemoto et al [26]	Interview	Interviewer	Upper body	Speech	Speech, behavior	Scripted
Bresó et al [27]	CBT	Counselor	Head-only	Speech	Speech, behavior	Rule-based
Takemoto et al [28]	Interview	Interviewer	Upper body	Speech	Speech, behavior	Scripted
DeVault et al [29]	Interview	Interviewer	Full body	Speech	Speech, behavior	Wizard of Oz
Kocur et al [32]	Avatar Therapy	Actor	Head-only	Speech	Speech, behavior	Wizard of Oz
Burton et al [33]	PHQ-9, CBT	Facilitator	Upper body	Text and speech	Speech, behavior	Scripted
Shamekhi et al [34]	Psychoeducation	Educator	Upper body	Touch	Text, speech, behavior	Scripted
Halim et al [35]	CFT	Actor	Full body	Speech	Speech, behavior	Recorded playback
Halim et al [36]	Psychoeducation	Educator	Full body	Touch	Text, speech, behavior	Scripted
Ring et al [37]	CBT	Counselor	Upper body	Text, speech, and video	Text, speech, behavior	Rule-based
Shamekhi et al [38]	Psychoeducation	Educator	Upper body	Touch	Text, speech, behavior	Scripted
Philip et al [39]	Interview	Interviewer	Upper body	Speech	Speech, behavior	Scripted
Ashrafi et al [30]	Avatar Therapy	Actor	Upper body	Speech	Speech, behavior	Scripted
Hidding et al [40]	CBT	Actor	Full body	Speech	Speech, behavior	Wizard of Oz
Wu et al [31]	Interview	Interviewer	Upper body	Speech	Speech, behavior	Scripted

a PHQ-9: Patient Health Questionnaire-9.

b CBT: cognitive behavioral therapy.

c CFT: compassion-focused therapy.

d LiCBT: low-intensity cognitive behavioral therapy.

## Results

### Summary of Selected Literature

**Figure 1.**
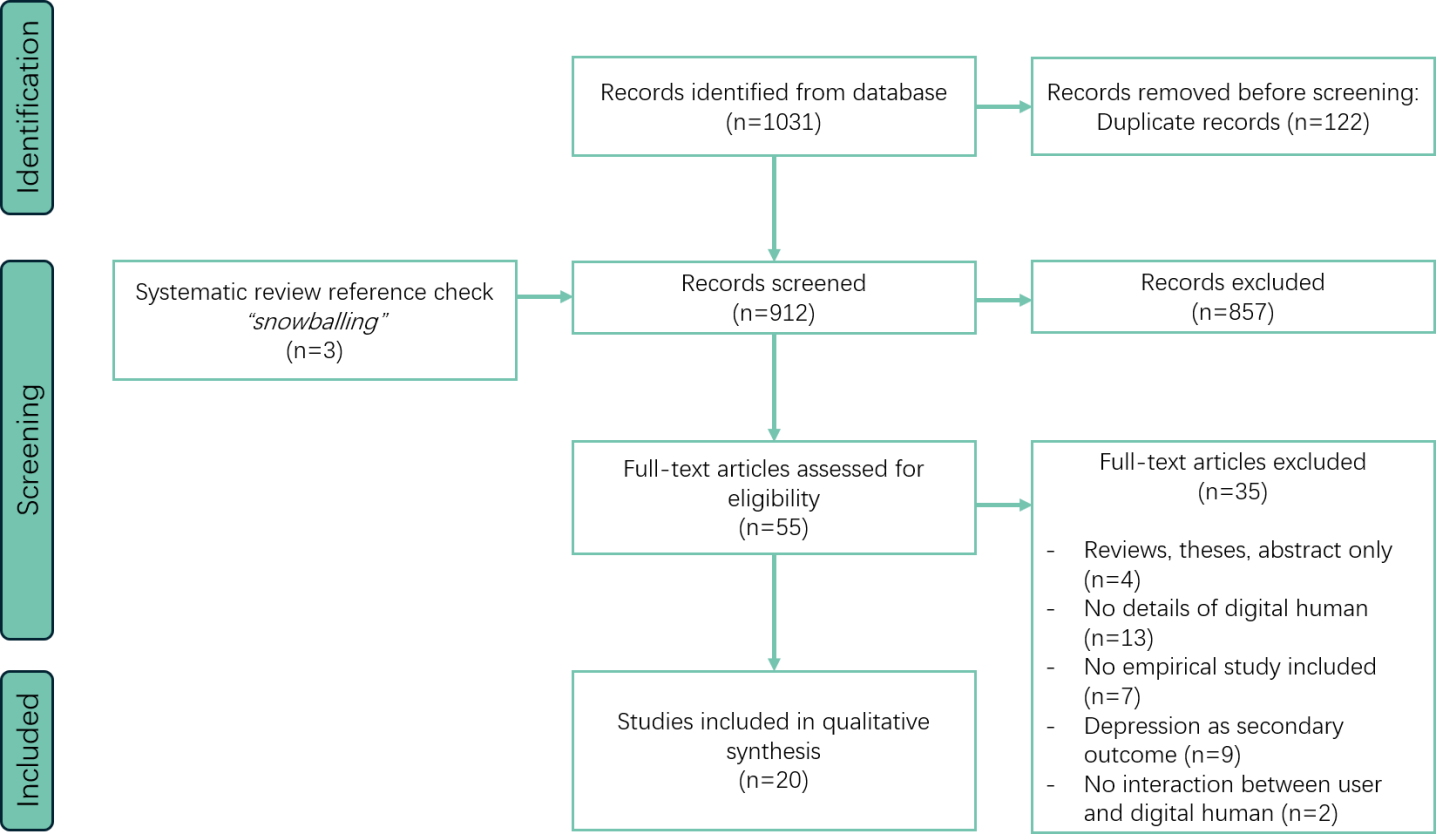
Literature screening and selection diagram following PRISMA guidelines. PRISMA: Preferred Reporting Items for Systematic Reviews and Meta-Analyses.

We completed the literature search in February 2025, and after the assessment stage, a total of 20 articles [21-40] were selected in this review, as detailed in Table 1. The detailed inclusion and exclusion criteria for studies at each phase of our review are visually represented in the PRISMA flow diagram (Figure 1).

The selected papers were published between 2010 and 2024, with those from the past 4 years accounting for 45% of the total (n=9) [22,24,26,28,30-32,35,40]. Of these, 10 papers (50%) [26-28,31-33,35,36,39,40] were presented in peer-reviewed journals, while the remaining articles were published in conference proceedings (n=10, 50%) [21-25,29,30,34,37,38].

More than half of the studies were conducted in research laboratory settings (n=12, 60%) [21,22,24,26-31,35,37,40], 5 experiments (25%) [23,25,33,34,38] took place in the participants’ everyday environments, and 3 (15%) [32,36,39] were conducted in clinical settings. Regarding participant numbers, 11 studies (55%) [23-26,28-30,33-35,37] involved fewer than 50 participants, 5 studies (25%) [21,22,27,32,40] had between 50 and 100 participants, and 4 studies (20%) [31,36,38,39] included more than 100 participants. Of these 4 studies with over 100 participants, 2 [36,39] were conducted in clinical environments, 1 [38] took place in an everyday environment, and 1 [31] in a laboratory setting.

In all the studies, those aimed at assessing depression accounted for 35% (n=7) [21,22,26,28,29,31,39], while studies focused on interventions constituted 55% (n=11) [24,25,27,30,32,34-38,40]. The remaining 2 studies [23,33], accounting for 10%, included both assessment and intervention.

### Summary of Support Services With Digital Human

We analyzed the selected studies primarily focusing on 3 factors: the protocols used, the roles played by digital humans within the service, and the experimental findings. Regarding protocols, commonly used assessment tools include questionnaires and interviews, as well as specially designed computer-based interactive tasks. Additionally, therapeutic approaches, such as psychoeducation, CBT [41], and CFT [42], have also been used. The roles of digital humans were primarily as interviewers, facilitators, and educators, actively interacting with participants. Notably, in the study by Baghaei et al [24], the digital human was designed as an actor, requiring proactive interaction from the participants. The findings from these experiments focused on 2 main areas—the usability and acceptability of digital humans in health care applications, and the potential for integrating digital humans into various protocols.

### Applied Protocol

#### Assessment

The applied protocol in assessment can be mainly divided into 3 categories—questionnaire, interview, and computer-based interactive task.

The first category is questionnaires, accounting for 3 of the studies [21,23,33]. In this category, the digital human will ask users the questionnaire questions one by one and collect their responses. Only answers that correspond to existing options will be collected; otherwise, the digital human will continue asking the user until obtaining a usable answer. The questionnaires that were used include PHQ-9 [43] and Center for Epidemiologic Studies Depression Scale - Visual Analog Scale - Very Short version (CES-D-VAS-VS) [44]. PHQ-9 is a brief self-report tool that consists of 9 items, each of which is scored on a scale from 0 (not at all) to 3 (nearly every day), which are directly derived from the diagnostic criteria for major depressive disorder in the *DSM*. The CES-D-VAS-VS is an adaptation of the traditional Center for Epidemiologic Studies - Depression Scale, which is a 20-item questionnaire used to measure depressive symptoms in the general population. The CES-D-VAS-VS incorporates a visual analog scale, enhancing its sensitivity by allowing patients to mark their symptom severity along a continuum.

The second category is assessments through interviews, accounting for 5 of the studies [26,28,29,31,39]. As an essential tool in depression assessment, interviews are commonly used in clinical settings where a nuanced understanding of the patient’s condition is crucial [45]. In the 5 studies that used interviews for depression assessment, 2 [26,28] focused on interviewing about negative topics, such as war and loneliness. These topics were chosen because they significantly impact vocal, visual, and verbal features, which are critical in detecting depression. The study by DeVault et al [29] based its interview content on observations from face-to-face interviews conducted in a clinical setting. This approach aimed to mimic real-life interactions and assess how patients express their symptoms naturally. Wu et al [31], through collaboration with clinicians, designed 93 questions about depression, anxiety, hypomania, and family relationships. This series of questions was specifically designed to vary depending on positive, negative, and neutral emotions. The study by Philip et al [39] structured its interview around the *DSM-5* [46] criteria for major depressive disorder. This method ensured that the interviews were comprehensive and aligned with established diagnostic standards, facilitating a more systematic approach to identifying depressive symptoms based on the latest psychiatric guidelines.

A computer-based interactive task was only used in 1 study [22]. In this study, researchers and mental health experts collaboratively designed 4 different types of tasks—mimicking, dyadic interaction, digital treatment, and psychometric. The design of these tasks aimed to be sufficiently engaging to prompt use without the need for physician guidance, executable on mobile devices, and effective at eliciting behavioral traits relevant to digitally assess mental health.

#### Intervention

There are a total of 13 (65%) studies focusing on the intervention phase. The therapeutic methods involved include CBT [23,25,27,33,37,40,47], psychoeducation [34,36,38], CFT [24,35], and avatar therapy [30,32].

CBT [48], as one of the most common therapy approaches in studies, aimed at teaching individuals how to recognize negative patterns of thought, evaluate their validity, and replace them with healthier ways of thinking. Among all the studies that applied CBT, they mainly focused on cognitive restructuring [49] and behavioral activation techniques [50]. In cognitive restructuring, participants need to recognize negative thoughts related to a recent event and are then encouraged to contemplate a more positive interpretation, and behavioral activation is a CBT technique that motivates participants to undertake avoided activities and engage in tasks that bring pleasure and accomplishment. Luerssen and Hawke [25] applied low-intensity CBT [51] in their study. Compared with traditional CBT, low-intensity CBT involves simpler interventions that can be delivered by practitioners who are not necessarily clinical psychologists and is more standardized, using general strategies that apply broadly to everyone with similar symptoms, while CBT is highly personalized and involves developing a specific therapeutic strategy for each patient’s unique problems and needs.

Psychoeducation is a fundamental component of depression intervention that involves educating individuals about depression as a disorder, including its symptoms, causes, and treatment options [52]. In the studies by Shamekhi et al [34,38], the content of psychoeducation primarily focuses on the management of nutrition, physical activity, pain, stress, sleep, and depression and includes guiding patients through practice sessions, such as meditation and yoga. Meanwhile, in the study by Bickmore et al [36], the educational content focused on the postdischarge self-care regimen, including medications, follow-up appointments, exercise and diet regimens, and pending laboratory tests.

In the remaining 4 studies, 2 [24,35] used CFT [42], a psychological approach designed to promote mental and emotional healing by encouraging individuals to develop compassion for themselves and others. The other 2 studies [30,32] used avatar therapy, where therapists interact with clients using a computer-generated avatar. Ashrafi et al [30] conducted a computer-assisted avatar-based treatment for dysfunctional beliefs (CAT-DB), which uses an avatar to help patients engage in dialogue with their dysfunctional beliefs and confront them.

### Role of Digital Human

In the selected studies, digital humans assumed 5 different roles—interviewer [21,26,28,29,31,39], facilitator [22,23,33], counselor [25,27,37], educator [34,36,38], and actor [24,30,32,35,40]. The interviewer primarily appears in assessment-type studies, where their main task is to present predesigned questions to participants and await their responses. Counselors also engage in conversation with participants, but their dialogues focus more on therapeutic interactions. For example, in the study by Ring et al [37], the digital human serves as a counselor, providing the first counseling session in a CBT intervention. Educators, designated specifically for psychoeducation, are responsible for conveying specific learning material to participants through visual and verbal means and assisting them in reviewing previously learned content. Facilitators focus on assisting participants in achieving specific goals requested by the system by providing task instructions and confirming task completion. Actor is a unique role compared with others; digital humans play specific characters to facilitate psychotherapy. In the study by Baghaei et al [24], the digital human acts in a state of negative emotion (angry, crying, etc) to elicit compassion from participants. In another study by Kocur et al [32], the digital human represents a human entity that continually expresses dysfunctional beliefs to participants, such as “You have to be perfect.”

### Feasibility and Effectiveness of Digital Human

#### Assessment

In studies applying digital humans to assessment, digital humans have been proven to be as effective in conducting interviews as showing real human videos [26,28] and filling out self- report questionnaires [21]. In the studies by DeVault et al [29] and Philip et al [39], data collected after interviews with digital humans were analyzed, and they concluded that these data have the potential to support automatic depression assessment based on dialogue systems involving digital humans and humans. Furthermore, in the experiment by Egede et al [22], it was found that participants exhibited greater body movements and more intense facial expressions under the guidance of a digital human (compared with text-only mode), which also supports the feasibility of using digital humans for automatic depression assessment.

#### Intervention

Many experimental results that apply digital humans in interventions have highlighted the potential for digital humans to establish positive relationships with participants. This includes factors such as positive responses to participants’ emotional expressions, customizable appearances, nonjudgmental expressions, and good patience. This personal relationship can also lead to better acceptance and attitudes toward digital humans from participants. Several articles reported that digital humans made a positive impression on participants. In the experiment by Bickmore et al [36], 76% of participants reported that their experience talking with a digital human was better than their interactions with health providers, and they were more inclined to choose digital humans as their channel for receiving psychoeducation in the future. Additionally, several articles indicated a positive impact of digital humans on therapeutic outcomes. For example, in the experiment by Kocur et al [32], they compared the results of participants receiving treatment as usual (TAU) with those receiving CAT-DB+TAU and found significant differences in the reduction of depressive symptoms in the group who received CAT-DB+TAU.

### Design of Digital Human

#### Overview

The design of digital humans across various studies showcases a diverse range of models, display devices, input or output modalities, and back-end intelligence, reflecting the adaptability of virtual characters to different applications. Based on the description of digital humans in the studies, we identified that the digital human designs in 3 studies [23,26,34] appeared repeatedly. We chose to retain the literature with more detailed descriptions of the digital human designs. After eliminating these duplicates, 17 unique digital human designs were included in the results.

#### Digital Human Presentation

**Figure 2.**
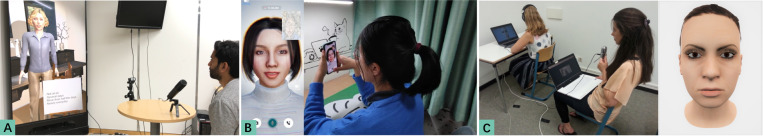
Example of digital human: (A) full body [21], (B) upper body [31], and (C) head-only [32].

In terms of body visibility, there are 7 full-body, 8 upper-body (head to chest), and 2 head-only digital humans (Figure 2). After analyzing the use cases for each digital human, we found that on mobile devices (such as phones and tablets), half-body is a more common setting [31,37], as this aligns better with how we typically see human bodies on these devices. The choice of full body is usually driven by device or scenario requirements. For example, digital humans in virtual reality (VR) all use full-body representations [24,35,40], and in the study by Stratou et al [53], they displayed a full-body digital human in larger screens to simulate the feeling of conversing with real people in reality. As mentioned by Bresó et al [27], they chose head-only because focusing on facial expressions makes it easier to convey different emotions and gives the digital human a higher degree of realism, which is a key issue in their study design. Regarding style design, the appearance of 2 digital humans leaned more toward a cartoon-like style [25,28]. Luerssen and Hawke [25] mentioned that their choice of cartoon-like style was to avoid the uncanny valley effect, while the rest of the digital humans adopted a more realistic style.

#### Input and Output Modality

The majority of digital humans (n=15, 88.2%) support natural language input, with 2 studies [36,38] relying on interface touch input. Correspondingly, all digital humans support speech synthesis and audio output, but only 5 digital humans are capable of delivering empathy narration [22,27,29,30,35]. All digital humans exhibit lip movements while speaking, along with other bodily actions, such as blinking [28] and nodding [29]. Due to their presence in a 3D environment, the digital humans in the studies by Baghaei et al [24], Halim et al [35], and Wu et al [31] are capable of a wider range of actions, including walking, crying, showing anger, and more.

#### Back-End Intelligence

This scoping review identifies 4 types of back-end intelligence used in the development of digital humans—Scripted Systems, Rule-Based Systems, Wizard of Oz, and Recorded Playback. Each approach offers unique strengths and applications, contributing to the diverse landscape of digital human technology.

Scripted systems operate based on predetermined scripts that define the digital human’s behavior and responses in a linear, predictable manner. This method is highly effective in scenarios where interactions are straightforward and the range of possible user inputs is limited. Among the 17 digital humans, more than half (n=10, 58.8%) used a scripted back end to control their behavior. For the digital human who used a scripted back end, the scenarios they face have standard dialogue processes, such as interviews with predefined topics [26,31], and tasks with specific instructions [22].

Rule-based systems are characterized by a set of predefined rules that guide the digital human’s decision-making processes. These systems offer greater flexibility compared with scripted systems, as they can dynamically adapt to a wider range of user inputs through conditional logic. Furthermore, 4 digital humans use a rule-based dialogue system, with 3 digital humans acting as counselors in the studies [25,27,37], and 1 as an interviewer [21].

The Wizard of Oz [54] technique involves a human operator who controls the digital human’s actions and responses in real-time, unbeknownst to the user. This method is commonly used in experimental and prototyping phases, allowing researchers to simulate the capabilities of a fully autonomous digital human before the underlying technology is fully developed. The digital humans in the experiments conducted by DeVault et al [29], Kocur et al [32], and Hidding et al [40] are directly controlled by professionals (certified counselors) to ensure participant safety and provide an experience closer to interacting with a real person.

Notably, in the experiment by Halim et al [35], the behavior of the digital human is derived from the participants’ actions, a method called Recorded Playback. In this experiment, participants are required to show compassion to the digital human in the first stage, and the language and actions of the participants are recorded by the system. In the second stage, these recorded behaviors are performed by the digital human.

## Discussion

### Principal Findings

#### RQ 1: Digital Human Replication of Roles in Supporting Services

The aim of this scoping review was to identify the existing evidence on using digital humans to assist in assessment for depression and delivering interventions, and to unfold the design choices of these digital humans. We have found that many studies designed digital humans’ purposes to be similar to certain roles in existing services. For example, a counselor conducting assessment through interviews [31], a therapist providing CBT session [27], and a nurse giving necessary information to patients [36]. The results of these studies can be considered preliminary affirmations of the effectiveness of digital humans in these roles.

Advantages of digital humans: Compared with real humans, digital humans have significant advantages in accessibility and availability, as they are essentially software programs. This means they can operate around the clock without the constraints of human limitations such as fatigue, working hours, or geographic location. This 24×7 availability ensures that support can be provided to users whenever they need it, which is particularly beneficial in emergency situations or for individuals in different time zones. Additionally, applying digital humans in the field of psychotherapy has a special advantage—the low social stigma caused by interaction. In selected studies, 2 articles [21,36] mentioned that in experimental interviews comparing interactions with digital humans and health providers (therapists or nurses), several participants felt more relaxed and safe communicating with the digital human. This advantage has been proven by the research by Lucas et al [55], which demonstrated that participants were more willing to disclose emotionally sensitive information, including expressing sadness more intensely, when they were interviewed by a digital human and believed the interviewer was a computer rather than a human. Another work by Loveys et al [56] indicates that the emotional expressiveness of digital humans, such as using an emotional voice, can enhance participants’ comfort and emotional responses during interactions. These studies suggest that digital humans can create an environment where individuals feel more comfortable and less anxious when disclosing. Furthermore, the consistency and unbiased nature of digital humans ensure that all users receive the same level of care and attention, free from the potential biases or variability that can come with human providers. This standardization can be particularly important in ensuring equitable access to high-quality support services.

Designed for real-life scenarios: In the design of these digital humans, a key design choice has been to ensure that the digital human’s appearance and behavior align with the local cultural context. For example, Takemoto et al [26] specifically mentioned that they designed the digital human’s appearance based on the local population’s characteristics. In the study by Wolters et al [23], besides the appearance, the digital human was given a Scottish accent to resonate more effectively with participants from that region. Based on previous research, such culturally sensitive design decisions can significantly enhance participants’ comfort and engagement, potentially leading to more effective therapeutic outcomes [57,58]. Moreover, the input modality is another vital consideration. While speech is often the natural and preferred input method of digital humans, some studies, particularly those conducted in noisy environments like hospitals [36], have opted for touch as the primary mode of interaction. This choice underscores the importance of usability in designing digital humans, emphasizing that how users interact with these entities must be carefully tailored to the specific context in which they are deployed.

Leveraging the advantages of digital humans, we believe that their potential can be extended to a wider range of roles to assist in depression assessment and intervention, especially in helping clients reduce social stigma. For instance, in face-to-face therapy, some clients may bring a support person to the session to help alleviate their anxiety. A digital human can replicate this support function by providing a nonjudgmental and consistent presence, helping clients feel more comfortable and less anxious during their sessions. Another promising role for digital humans is that of a peer specialist. Peer support interventions have been shown to be effective in treating depression, as evidenced by numerous studies [59]. Peer specialists share their own experiences to help clients feel understood and to encourage them to disclose more during the intervention. A digital peer specialist can fulfill this role by simulating these supportive interactions, providing empathetic responses, and sharing relatable experiences that resonate with clients.

#### RQ2: Design Considerations for Digital Human Implementation

Digital human design choices reflect careful consideration of technological constraints, therapeutic goals, and user contexts. The analysis reveals systematic patterns in how researchers approach fundamental design decisions, from visual appearance to underlying intelligence architectures, with each choice serving specific therapeutic and technical purposes.

Body visibility decisions appear strategically aligned with platform capabilities and therapeutic objectives. Full-body representations dominate VR applications where spatial presence is paramount, while upper-body presentations are preferred for mobile devices where screen space is limited. Head-only implementations are chosen specifically when facial expression fidelity is critical for conveying emotions or avoiding uncanny valley effects [27,32]. This distribution suggests that successful digital human design requires careful consideration of platform affordances and their alignment with therapeutic goals. The predominance of realistic over cartoon-like styling (15 of 17 designs) suggests a preference for human-like appearance that supports therapeutic credibility and user engagement. However, some researchers deliberately chose cartoon styling to avoid uncanny valley effects while maintaining user comfort, indicating that appearance realism must be balanced against potential negative user reactions. Cultural sensitivity emerges as a critical design consideration, with researchers adapting appearance characteristics, accents, and interaction styles to match local population demographics and cultural expectations. These adaptations demonstrate awareness that effective therapeutic relationships require cultural alignment and familiarity.

Interaction modality design reveals sophisticated approaches to creating natural, accessible interfaces. The overwhelming preference for speech-based input (88.2% of implementations) reflects both user expectations for natural conversation and the technological maturity of speech recognition systems. Hence, the conversation ability is one of the main factors that affect closeness perceptions on digital humans [60]. The selective use of touch input in specific contexts, particularly noisy hospital environments, demonstrates adaptive design thinking that prioritizes usability over technological sophistication. This flexibility suggests that effective digital human systems must accommodate diverse deployment contexts and user needs.

Output modality design shows varying levels of sophistication in multimodal communication. While all systems provide speech synthesis and basic behavioral animations, the implementation of empathy narration in only 5 systems suggests that this remains a challenging but valuable design goal. The expanded behavioral repertoires available in 3D environments, including walking, crying, and showing anger, highlight how platform capabilities directly influence design possibilities and therapeutic potential. These variations indicate that output sophistication should align with both technical capabilities and therapeutic requirements.

The distribution of back-end intelligence approaches reveals important insights about current technological capabilities and design priorities. Scripted systems (10/17, 58.8%) dominate in contexts requiring predictable, standardized interactions, particularly for assessment protocols and structured educational content. Rule-based systems (4/17, 23.5%) appear primarily in counseling contexts where greater conversational flexibility is needed while maintaining therapeutic boundaries. This distribution suggests that different therapeutic contexts require different levels of conversational sophistication and autonomy. The continued use of Wizard of Oz techniques (3/17, 17.6%) in recent studies indicates that fully autonomous digital humans may not yet be ready for complex therapeutic interactions, particularly those requiring real-time clinical judgment. This approach allows researchers to explore the therapeutic potential of digital humans while ensuring participant safety and intervention quality. The innovative use of recorded playback in CFT [35] demonstrates how back-end intelligence can be tailored to specific therapeutic mechanisms, using participants’ own recorded behaviors to create personalized therapeutic content.

The design of digital humans for depression support requires balancing multiple factors: Visual appearance needs to be culturally adapted to the target population and matched to platform capabilities, and within these constraints, should be realistic enough for credibility while avoiding uncanny valley effects. Interaction design should prioritize natural speech input where feasible but adapt to environmental constraints. Back-end intelligence approaches must be selected based on therapeutic context, with scripted systems providing safety and standardization for assessments, while rule-based and Wizard of Oz techniques enable more flexible therapeutic interactions. Ultimately, successful digital human implementation depends on aligning design choices with specific therapeutic goals, technological capabilities, and user needs rather than pursuing technological sophistication for its own sake.

#### Digital Human in Building Therapeutic Alliance

The therapeutic alliance, characterized by a collaborative and trusting relationship between a health provider and a client, is a cornerstone of effective psychotherapy [61]. Traditionally, this alliance is built through face-to-face interactions, where empathy, understanding, and mutual respect foster a sense of safety and connection. With the advent of digital humans in therapeutic settings, there is a growing interest in understanding how these virtual entities can contribute to building and maintaining a therapeutic alliance [62].

In this scoping review, multiple studies [23,36,37,39] have demonstrated the potential of digital humans to establish trustworthy and comfortable relationships with clients. These findings underscore the viability of digital humans as supportive agents in mental health care. Several key factors contribute to the successful establishment of therapeutic alliances with digital humans. First is empathy and emotional responsiveness. The ability to empathize is crucial in building a therapeutic alliance [63]. Digital humans, as embodied virtual agents, can combine facial expressions, body movements, and emotional narration to convey emotions accurately. In the study by Ring et al [37], participants felt that their feelings were understood because the digital human provided appropriate feedback. Similar results were observed in the experiment by Philip et al [39], where participants also reported a sense of being understood. These findings highlight the importance of empathetic interactions in fostering a strong therapeutic alliance. Another critical factor is the nonjudgmental presence of digital humans. This presence is created through the careful design and control of the content expressed by the digital human. The interactive content of digital humans applied in all of the 17 included studies was designed or evaluated by experts before being applied in experiments. Although this approach limits the range of user interactions, it ensures the safety and appropriateness of the interactions. By providing a nonjudgmental and safe space, digital humans can help clients feel more comfortable and open, thereby strengthening the therapeutic alliance. In addition, compared to face-to-face interactions, digital humans offer customizable support tailored to the individual’s personality and needs. This personalized attention can make people with depression feel valued and understood, increasing their motivation to engage in therapy and fostering a deeper sense of trust.

Digital humans have demonstrated significant potential in building therapeutic alliances with individuals experiencing depression. By providing empathetic, consistent, and personalized support, digital humans can help overcome barriers related to stigma, availability, and accessibility.

#### Ethical Considerations and Potential Risks

While this scoping review demonstrates the promising potential of digital humans in depression management, implementation raises critical ethical concerns requiring careful attention. Privacy and data security present immediate risks, as digital human systems collect highly sensitive information, including verbal responses [35], behavioral patterns [39], and potentially video or audio recordings during vulnerable therapeutic moments [37]. Many reviewed studies provided limited detail about data management practices, yet these systems often rely on cloud-based infrastructure and third-party services that create multiple exposure points for sensitive mental health data. Biometric information, such as voice recordings and facial expressions, is inherently difficult to anonymize and could potentially reidentify individuals even after traditional identifiers are removed. Future implementations should ensure robust encryption, secure storage protocols compliant with relevant regulations, transparent disclosure of data practices, and clear policies regarding third-party access and data retention.

The accessibility advantages of digital humans paradoxically create risks of overreliance and inappropriate use. Recent news has shown that individuals may incorrectly view these tools as complete replacements for human therapists rather than complementary supports [64], potentially delaying access to necessary human intervention for severe depression, suicidal ideation, or complex presentations. Current autonomous systems have limited capacity to recognize and appropriately respond to acute crises, and users may form parasocial relationships with digital humans without recognizing fundamental limitations compared with human therapeutic relationships. Clear communication about system boundaries is essential, alongside robust escalation mechanisms to human providers when needed. Additionally, questions of accountability remain unresolved—when adverse outcomes occur, responsibility distributed among developers, health care providers, and users requires clear frameworks that protect vulnerable populations while enabling innovation. Equity and access concerns threaten to undermine the democratizing potential of digital humans. Implementation requires technological access (appropriate devices, reliable internet, and digital literacy) that populations at risk for depression—including low-income individuals, older adult persons, and those in rural areas—may lack. Only 25% (5/20) [23,25,33,34,38] of reviewed studies occurred in everyday environments, suggesting limited real-world accessibility evidence. Without deliberate policy intervention ensuring equitable access through public health systems and subsidized programs, these technologies risk becoming available primarily through private markets, widening rather than closing mental health care gaps.

Moving forward, ethical implementation requires privacy-by-design approaches with comprehensive data protection, transparent communication of system limitations and appropriate use cases, inclusive development ensuring diverse representation in training data and validation studies, and hybrid care models positioning digital humans as adjuncts rather than replacements for human providers. Regulatory frameworks must establish appropriate validation standards and postmarket surveillance requirements, while sustainable funding models must prioritize access for underserved populations. As this technology advances beyond proof-of-concept demonstrations, the field should evaluate not only what digital humans can do, but what they should do, ensuring that the pursuit of innovation maintains focus on user well-being, autonomy, safety, and equitable access across all populations who might benefit from mental health support.

### Suggestions for Future Research

A total of 3 promising research opportunities emerged from this review that offer significant potential to advance the field of digital humans in depression management.

First, the current technological landscape reveals substantial opportunities for enhancement that could transform therapeutic capabilities. Future research would benefit from exploring the integration of physiological monitoring (heart rate variability, galvanic skin response, and eye tracking) with digital human systems to enable real-time emotional state detection and adaptive responses. Given that current systems rely predominantly on scripted or rule-based approaches, there is potential for incorporating enhanced natural language processing and emotional artificial intelligence capabilities while maintaining therapeutic safety standards. Cross-platform optimization research presents exciting opportunities, as current use of digital humans has appeared across mobile platforms (smartphones and tablets) [25,38], stationary platforms (large display systems and desktop monitors) [21,27], and VR headsets [35,40], suggesting the need for thoughtful platform-specific design guidelines and seamless integration across devices to maintain therapeutic continuity.

Building on the demonstrated success of digital humans in traditional clinical roles, research could explore innovative therapeutic applications that leverage their unique capabilities. The encouraging success of digital humans as “actors” representing emotions and thoughts suggests promising potential for novel approaches, including digital peer specialists who can share lived experiences, family therapy facilitators who manage complex group dynamics, and group intervention leaders who ensure equitable participation. Prevention and early intervention applications present valuable research opportunities, as current studies focus primarily on individuals already experiencing depression rather than exploring applications with at-risk populations, such as adolescents, caregivers, or individuals with chronic medical conditions. Research could also investigate how digital humans can serve as effective mediators between counselors and clients, facilitating communication and building trust through their demonstrated ability to reduce stigma and encourage self-disclosure.

Finally, research would greatly benefit from investigating how digital humans can be effectively integrated into existing health care workflows, including exploring optimal combinations of digital and human therapist interactions, developing smooth hand-off protocols between digital and human providers, and establishing data-sharing mechanisms that maintain continuity of care. Cost-effectiveness analyses offer valuable opportunities to demonstrate the economic benefits of digital human interventions compared with traditional care models, while implementation research could systematically explore facilitators and address barriers to adoption across diverse clinical settings, from large hospital systems to community health centers.

Besides these research opportunities, we also recognize the need for concrete methodological guidance to advance the field beyond its current proof-of-concept stage. Future studies should use adequate sample sizes appropriate to their RQs: proof-of-concept studies should include a minimum of 30‐50 participants for preliminary feasibility assessment, while comparative effectiveness trials require 100‐150 per arm to detect medium effect sizes with adequate power. Researchers should adopt standardized outcome measures to enable meta-analyses and cross-study comparisons, including PHQ-9 or Beck Depression Inventory-II for depression symptom severity, the Working Alliance Inventory adapted for digital humans to assess therapeutic alliance, the System Usability Scale for usability evaluation, and systematic adverse event monitoring. Additionally, we suggest that future research include more detailed documentation of the digital human design process to enhance transparency and replicability. As noted in our limitations, most reviewed studies provided only final design descriptions without elaborating on the underlying rationale, iterative decisions, or user-centered design methodologies used.

### Practical Implications

#### User-Centered Design Principles

The review highlights that effective digital human systems are grounded in collaborative design processes between clinicians and developers. To ensure therapeutic relevance and usability:

- Clinicians should be involved early to define therapeutic goals, patient needs, and appropriate boundaries.
- Developers should guide feasibility discussions and apply user experience principles tailored to mental health contexts.
- Ongoing dialogue should promote mutual understanding—clinicians educate on therapeutic aspects of the project, while developers clarify what current technologies can and cannot achieve.

This interdisciplinary co-design ensures that systems are both clinically sound and technically viable.

#### Ethical Implementation Guidelines

The deployment of digital humans in mental health care raises important ethical considerations that require careful attention during study. Based on the review findings, we suggest:

- Study protocols should prioritize transparency about the interactions between participants and digital humans, especially the data collection practices.
- Participants should be clearly informed about the capabilities and limitations of digital human systems, including their role as therapeutic tools rather than replacements for human care.
- Implementation frameworks should establish clear boundaries and expectations from the outset—clinicians define appropriate therapeutic limits and user relationship parameters, while developers implement technical safeguards that support these ethical boundaries.

#### Integration Into Clinical Workflows

Successful implementation of digital humans in depression care requires strategic planning across multiple dimensions:

- Design the digital human’s appearance and language to reflect target user population characteristics (eg, facial features, accent, and ethnic background).
- Assess the physical environment where deployment will occur and select interaction modalities appropriate to the setting (eg, touch-based input for noisy environments and speech input for quiet, private spaces).
- Start with well-defined tasks where scripted systems have proven effective: standardized questionnaire administration (PHQ-9), protocol-based interviews, and psychoeducational content delivery.
- Provide training for clinical staff on system capabilities, limitations, and appropriate use cases.
- Implement feedback mechanisms to continuously improve system performance based on clinician and patient input.

### Limitations

This scoping review has some limitations. To make our review feasible, we used a relatively narrow literature search approach, which may have introduced selection bias. The restriction to studies published in English could have excluded relevant research, and due to the various terms used to describe digital humans in the literature, our search terms might not have covered all relevant papers.

The studies included in this review exhibited considerable heterogeneity in terms of design, methodology, and intervention protocols. This makes it challenging to draw definitive conclusions or directly compare outcomes across different studies. Critically, the evidence base is dominated by proof-of-concept studies and small-sample trials. Specifically, 55% of studies (n=11) [23-26,28-30,33-35,37] included fewer than 50 participants, with only 20% (n=4) [31,36,38,39] enrolling over 100 participants. This prevalence of small-scale exploratory research limits the statistical power and generalizability of findings. While these developmental studies provide valuable preliminary evidence, they are insufficient for making definitive claims about therapeutic efficacy. The differences in study designs, sample sizes, and measurement tools underscore that digital humans for depression management remain an emerging field requiring substantial methodological advancement before strong clinical recommendations can be made.

Another limitation identified in the included studies is the lack of detailed information on the design processes for digital humans in the reviewed studies. Most studies provided descriptions of the functions and appearances of digital humans but did not elaborate on the underlying design choices or rationales. This lack of transparency makes it difficult to understand how specific design decisions might have influenced study outcomes and limits our ability to evaluate the replicability and effectiveness of different digital human implementations. As with most systematic literature reviews, potential publication bias is also a concern, as unpublished studies or those with negative results might not have been included in this review.

### Conclusions

This scoping review of 20 studies systematically addressed how digital humans are used in depression management and their design considerations. Regarding usage (RQ1), digital humans demonstrate versatility across assessment (9/20, 45%) through questionnaire administration, interviews, and interactive tasks, and intervention (13/20, 65%) through CBT, psychoeducation, and innovative therapies, assuming roles as interviewers, facilitators, counselors, educators, and actors. For design considerations (RQ2), successful implementations strategically align appearance with platform capabilities (full-body for VR and upper-body for mobile), prioritize realistic styling and cultural sensitivity, use speech-based input (15/17, 88.2%), and use back-end intelligence ranging from scripted (10/17, 58.8%) to rule-based systems. The use of digital humans now stands at a juncture where it not only enables bidirectional conversations but also significantly enriches interactions within the domain of depression management. This advancement signals a paradigm shift toward fostering deeper connections, tailored assistance, and broadening the horizons of accessibility, surpassing what traditional therapeutic frameworks could offer. Our scoping review has charted the pioneering deployment of digital humans across the dual spectrums of assessment and intervention, casting a spotlight on their transformative potential to amplify the reach and resonance of mental health interventions.

## Supplementary material

10.2196/79954 Checklist 1 PRISMA-ScR checklist.

## Abbreviations

CAT-DB: computer-assisted avatar-based treatment for dysfunctional beliefs
CBT: cognitive behavioral therapy
CES-D-VAS-VS: Center for Epidemiologic Studies Depression Scale - Visual Analog Scale - Very Short version
CFT: compassion-focused therapy
DSM-5: Diagnostic and Statistical Manual of Mental Disorders, Fifth Edition
PHQ-9: Patient Health Questionnaire-9
PRISMA: Preferred Reporting Items for Systematic Reviews and Meta-Analyses
PRISMA-ScR: Preferred Reporting Items for Systematic Reviews and Meta-Analyses extension for Scoping Reviews
RQ: research question
TAU: treatment as usual
VR: virtual reality
